# Cortical and subcortical grey matter correlates of psychopathic traits in a Japanese community sample of young adults: sex and configurations of factors’ level matter!

**DOI:** 10.1093/cercor/bhac397

**Published:** 2022-10-27

**Authors:** Sally C Chester, Tatsuyoshi Ogawa, Maki Terao, Ryusuke Nakai, Nobuhito Abe, Stephane A De Brito

**Affiliations:** Centre for Human Brain Health, School of Psychology, University of Birmingham, Birmingham, UK; Division of Transdisciplinary Sciences, Graduate School of Advanced Science and Technology, Japan Advanced Institute of Science and Technology, Nomi, Ishikawa, Japan; Institute for the Future of Human Society, Kyoto University, Sakyo-ku, Kyoto, Kyoto, Japan; Institute for the Future of Human Society, Kyoto University, Sakyo-ku, Kyoto, Kyoto, Japan; Institute for the Future of Human Society, Kyoto University, Sakyo-ku, Kyoto, Kyoto, Japan; Centre for Human Brain Health, School of Psychology, University of Birmingham, Birmingham, UK

**Keywords:** gray matter volume, psychopathy, sex differences, surface-based morphometry, voxel-based morphometry

## Abstract

While neuroimaging research has examined the structural brain correlates of psychopathy predominantly in clinical/forensic male samples from western countries, much less is known about those correlates in non-western community samples. Here, structural magnetic resonance imaging data were analyzed using voxel- and surface-based morphometry to investigate the neuroanatomical correlates of psychopathic traits in a mixed-sex sample of 97 well-functioning Japanese adults (45 males, 21–39 years; M = 27, SD = 5.3). Psychopathic traits were assessed using the Self-Report Psychopathy Scale (SRP-SF; 4^th^ Edition). Multiple regression analysis showed greater *Factor 1* scores were associated with higher gyrification in the lingual gyrus, and gray matter volume in the anterior cingulate cortex and amygdala/hippocampus border. *Total psychopathy* and *Factor 1* scores interacted with sex to, respectively, predict cortical thickness in the precuneus and gyrification in the superior temporal gyrus. Finally, *Factor 1* and *Factor 2* traits interacted to predict gyrification in the posterior cingulate cortex*.* These preliminary data suggest that, while there may be commonalities in the loci of structural brain correlates of psychopathic traits in clinical/forensic and community samples, the nature of that association might be different (i.e. positive) and may vary according to sex and configurations of factors’ level.

## Introduction

Psychopathy is a personality disorder characterized by distinctive interpersonal and affective abnormalities and repeated disruptive and antisocial behaviors (Hare and Neumann 2009). Factor analytic work has shown that psychopathy is underpinned by two correlating higher order factors. Factor 1 relates to affective-interpersonal traits including callousness, egocentricity, lack of remorse or empathy, and manipulativeness while factor 2 indexes impulsive-antisocial traits such as impulsivity, stimulation seeking and criminal versatility (Hare and Neumann 2008; Hare and Neumann 2009) (More recently, authors have proposed that these factors be further delineated into four distinct facets: interpersonal (facet 1); affective (facet 2); erratic lifestyle (facet 3) and antisocial (facet 4) (Vitacco et al. 2005)). Individuals with the disorder are at high risk of poor clinical and behavioral outcomes including violence, criminality, and other externalizing psychopathology (Salekin et al. 1997). As such, identifying the neural basis of psychopathy is an important area of research that could have profound implications for the assessment and management of these individuals.

Voxel-based morphometry (VBM) and surface-based morphometry (SBM) are two commonly used automated whole-brain brain morphometry techniques used to analyze structural magnetic resonance imaging (sMRI) data. In VBM, structural images are normalized to the same stereotaxic space, segmented, modulated, and smoothed. Statistical analysis is performed on a voxel-by-voxel basis, either across the whole brain or regions of interest, to characterize and detect differences in gray matter volume (GMV) while controlling for global brain shape differences (Ashburner and Friston 2000). However, VBM results can be difficult to interpret as GMV is a composite measure underpinned by cortical thickness, surface area and folding patterns (e.g. local gyrification index or sulcus depth), or a combination of these measures (Panizzon et al. 2009). In SBM, surface-based metrics are extracted from a surface model of the cortex and a vertex-wide statistical analysis is performed (Landin-Romero et al. 2017). Combining these types of morphometry, as in the present study, can circumvent issues that have been raised regarding VBM (e.g. normalization procedures (Bookstein 2001)) and can provide distinct and complementary information about the neural correlates of a particular disorder allowing for a comprehensive neuroanatomical analysis.

Previous sMRI studies have identified several structural brain abnormalities in psychopathy. A recent literature review outlines GMV reductions in several areas of the prefrontal, temporal, and parietal cortices (precuneus and postcentral gyrus) as well as in limbic structures including the amygdala, hippocampus, cingulate (anterior, middle, and posterior subdivisions), and insular cortices (Johanson et al. 2020). Likewise, a recent meta-analysis of VBM studies found significantly reduced GMV within the left hemisphere in the dorsolateral prefrontal and orbitofrontal cortices (OFC) (De Brito et al. 2021b). Surface-based abnormalities have been identified in similar cortical and subcortical regions, including the temporal lobes and entire right hemisphere (Howner et al. 2012), left insula, dorsal anterior cingulate cortex, precentral gyrus, temporal pole, and inferior frontal gyrus (Ly et al. 2012), in which prisoners with psychopathy had a significantly thinner cortex than non-psychopaths. A more recent study investigating gyrification in a sample of 716 incarcerated males found negative associations between psychopathy scores and local gyrification index in the right midcingulate cortex, dorsomedial frontal cortex and right lateral superior parietal cortex (Miskovich et al. 2018). Similarly, data from community samples have identified significant gray matter thinning in the right prefrontal and temporal cortices (Yang et al. 2009a), left orbitofrontal cortex and bilateral anterior temporal cortex (Yang et al. 2011) along with structural differences in the amygdala (Yang et al. 2009b) in individuals with psychopathy compared to healthy controls.

Although some debate remains regarding the core features of the disorder (Hare and Neumann 2010), most etiological models of psychopathy allow for multiple facet configurations and severity levels (Sellbom and Drislane 2021). Indeed, factor analytic work performed on several commonly used psychopathy measures has provided support for the multifaceted structure of the disorder (Benning et al. 2003; Hill et al. 2004; Vitacco et al. 2005; Christian and Sellbom 2016). Accordingly, some authors have suggested that the two factors of psychopathy might be underpinned by different pathophysiological processes (Yang and Raine 2009) and emerging neuroimaging evidence supports this view.

For example, higher Factor 1 scores have been related to decreased GMV in the left supplementary motor area and middle inferior frontal gyrus (De Brito et al. 2021b), right rostral middle frontal region, pericalcarine region, hippocampus, caudate (Miglin et al. 2021), right inferior temporal gyrus (Cope et al. 2012), and amygdala (Contreras-Rodríguez et al. 2015). They have also been associated with gray matter thinning in the temporal lobes (Howner et al. 2012) and right temporal and frontal cortices (Yang et al. 2009a) and reduced gyrification in the right midcingulate cortex (MCC) (Miskovich et al. 2018). Positive associations between Factor 1 scores and gyrification were also identified within the left and right occipital cortex (Miskovich et al. 2018).

Higher Factor 2 scores have been shown to negatively correlate with caudal volume and regional gray matter in the left middle occipital gyrus (De Brito et al. 2021b) and right insula (Cope et al. 2012) and have been associated with reduced cortical thickness in the right frontal and parietal lobes, and bilateral temporal lobes (Howner et al. 2012). Positive correlations have also been found between Factor 2 scores and GMV within medial and lateral frontal cortices (Contreras-Rodríguez et al. 2015), supplementary motor area, basal ganglia regions, orbitofrontal cortex, and insula (Leutgeb et al. 2015).

Finally, to test the hypothesis that the two psychopathy factors may be differentially associated with and interactively related to cortical and subcortical GMV, Miglin et al. (2021) measured GMV and psychopathic traits in a mixed-gender sample (*n* = 156) recruited from communities with high rates of crime and found several factor-level interaction effects. Specifically, in the left lingual gyrus, as scores on Factor 2 increased, the association between GMV and Factor 1 scores became more positive. Furthermore, at low levels of Factor 2, Factor 1 was positively associated with GMV in two cortical clusters (peaking in the left superior parietal lobule and right supramarginal gyrus, respectively) and bilateral amygdala and hippocampus, whereas at high levels of Factor 2, the opposite pattern was observed. This provides evidence that those high on both psychopathy Factors (1 and 2) may be neurobiologically different to those that score high on only one factor. Taken together, these results suggest that the two factors of psychopathy might be associated with distinct structural brain correlates and that interaction effects between factors might clarify the nature of the associations between psychopathy facets and sMRI correlates.

Importantly, the current sMRI literature on psychopathy suffers from two notable sampling limitations, namely an over-reliance on male samples from forensic and clinical populations and an absence of studies including non-Westerner populations. Indeed, while the pattern of core characteristics present in males, particularly interpersonal and affective dysfunctions, such as deficits in empathy and moral processing (Seara-Cardoso et al. 2013), can also be identified in females (Forouzan and Cooke 2005), there is a clear sex difference in the prevalence of the disorder (Beryl et al. 2014). Furthermore, several lines of evidence suggest that, across sexes, there may be significant differences in etiological pathways and psychological risk factors for psychopathy (Cale and Lilienfeld 2002; Falk et al. 2014).

There is also good evidence that psychopathy is a dimensional construct existing on a continuum (Guay et al. 2007; Neumann and Hare 2008; Haslam et al. 2020), but most of the neuroimaging research to date has been conducted on forensic and clinical samples representing the extreme presentation of the disorder, likely explained by the higher prevalence of the disorder in prison (~25%) vs. community (~1%) (De Brito et al. 2021a). However, neuroimaging studies on community samples are needed to establish if, to what extent, there is also continuity in the mechanisms underlying psychopathy (Koenigs et al. 2011; Seara-Cardoso and Viding 2015).

Results from functional and structural neuroimaging studies using community samples generally reflect the findings from forensic and clinical samples (Seara-Cardoso and Viding 2015). For example, higher psychopathy scores have been associated with decreased GMV in the left striatum and amygdala (Vieira et al. 2015) anterior insula, OFC, and secondary somatosensory cortex (Nummenmaa et al. 2021), which have also been implicated in offenders with psychopathy. Howner et al. (2012) also found, across participants (including offenders with psychopathy, autism spectrum disorder, and healthy controls), negative correlations between psychopathy scores and cortical thickness in the right and left temporal lobe, and as well as in the whole right hemisphere. Such findings suggest that there may be commonalities in the structural brain correlates of psychopathic traits in community and forensic samples.

Finally, a frequent criticism of behavioral science concerns the general use of samples drawn from Western, Educated, Industrialized, Rich and Democratic (WEIRD) nations (Henrich et al. 2010; Rad et al. 2018). Indeed, what we know about psychopathy is largely based upon studies conducted in Western nations and there has been a general paucity of systematic research relating to countries outside the United States and Europe. However, conceptualizations and operationalizations of disorders that are heavily influenced by Western individualistic psychological processes may be inadequate in capturing personality disorders in other socio-cultural contexts (Shou et al. 2021). This is of particular importance when considering psychopathic traits and the behaviors by which they manifest. Generally, the core behavioral characteristics of extreme personality disorders are conceptualized as those that deviate substantially from societal norms, thinking styles and associated behavior patterns (Shou et al. 2021). However, studies often fail to consider socio-cultural milieus and experiences that vary across nations or how cultural features may shape an individual’s concept of self and others (Triandis and Suh 2002; Heine and Buchtel 2009). Furthermore, research suggests cognitive functions, overall brain size and shape, neural activation patterns, hemispheric shape, and the volume of neural structures differ as a function of culture (Zilles et al. 2001; Hedden et al. 2008; Kitayama and Uskul 2011; Huang et al. 2019; Yu et al. 2019). Thus, such findings underscore the importance of investigating the neural correlates of psychopathy in non-western samples.

In the present study, we collected structural neuroimaging data and measured psychopathic traits in a sample of well-functioning male and female Japanese young adults. We analyzed the data using VBM and SBM to address the following aims: (i) identify the main effects of psychopathy, and its factors, on GMV across the whole brain and in key neural structures; (ii) explore the associations between surface-based characteristics and psychopathy, and its subcomponent traits, using measurements of cortical thickness and cortical folding (gyrification and sulcal depth); (iii) determine if sex influenced the strength or direction of such associations; and (iv) investigate interactive effects of psychopathy traits on GMV and surface-based characteristics.

## Materials and methods

### Participants

Ninety-seven (45 males) right-handed participants aged 21–39 years (M = 27, SD = 5.3) from the community were recruited from a Japanese work placement agency. All participants took part in standardized psychometric testing and neuroimaging carried out at Kyoto University, Japan. All involvement commenced with the full informed consent of participants and approval of the Ethics Committee at Kyoto University.

### Psychopathic traits assessment

Psychopathic traits were measured using the Self-Report Psychopathy Scale Fourth Edition (SRP-4) Short Form, which comprises 29-items and uses a five-point Likert scale that ranges from 1 (“strongly disagree”) to 5 (“strongly agree”). It provides a total score for overall psychopathy along with four sub-scale scores for each of the facets (antisocial behavior (ASB) (8 items, α = 0.63), callous affect (CA) (7 items, α = 0.67), interpersonal manipulation (IM) (7 items, α = 0.68), and erratic lifestyle (EL) (7 items, α = 0.82)). In the present study, responses on the callous affect and interpersonal manipulation sub-scales were summed to create a “Factor 1” score (14 items, α = 0.85) and responses on the antisocial behavior and erratic lifestyle sub-scales were summed to create a “Factor 2” score (15 items, α = 0.76). Research has found the SRP-4 and SRP-SF (short form) are strongly correlated (*r* = 0.96, *P* < 0.01) (Tew et al. 2015) meaning the short version provides a viable and efficient alternative to the full version. Yanagida et al. (2018) have examined the validity of the Japanese version of the SRP-SF in a community population. The study used 486 Japanese students to examine the factor structure of the SRP-SF and its relation to other common psychopathy scales. The four-factor structure of psychopathy was strongly replicated in a confirmatory factor analysis (factors: interpersonal manipulation, antisocial nature, cold emotions, and unstable lifestyle). Furthermore, significant positive correlations were demonstrated between the Japanese version of the SRP-SF and other validated measures of psychopathy. As such, the results of the study support the validity, and use of the SRP-SF in Japan.

### Structural MRI acquisition

Magnetic resonance imaging was performed at Kyoto University on a Siemens 3 T Verio scanner with a 32-channel head coil. A structural image was acquired for each participant using a T1 weighted Magnetization Prepared Rapid Acquisition Gradient Echo (MPRAGE) sequence (repetition time = 2,500 ms, echo time = 2.18 ms, flip angle = 8 degrees, field of view = 256 mm, voxel size 0.8 × 0.8 × 0.8 mm) yielding 224 sagittal slices. Duration of scan was 5 minutes and 22 seconds.

### MRI pre-processing

MRI scans were pre-processed and analyzed using the Computational Anatomy Toolbox (CAT12) within the Statistical Parametric Mapping 12 (SPM12) software running in MATLAB R2020b. The default pipeline and recommended settings according to the CAT12 manual were used. Specifically, images underwent bias correction, initial spatial registration, and were segmented into gray matter (GM), white matter (WM), and cerebral spinal fluid (CSF) using the SPM12 tissue probability maps (TPM). Images were normalized (warped and modulated) to a study-specific template using a Diffeomorphic Anatomical Registration Through Exponentiate Lie Algebra (DARTEL) algorithm. All scans were smoothed with an 8 mm full-width at half-maximum Gaussian kernel.

Total intracranial volume (TIV) was calculated by summing GM, WM, and CSF to be included as a covariate of no interest in subsequent statistical analysis. However, upon checking for design orthogonality, co-linearity was found between TIV and parameters of interest. As such, global scaling using the TIV values and mean TIV value was performed to proportionally scale the data according to individual TIV values as per the CAT12 manual.

Additional surface parameters (sulcal depth, thickness and gyrification index) were also extracted during the pre-processing stages using the default automated algorithms within the CAT12 settings. As recommended by the developers (Gaser et al. 2022, http://dbm.neuro.uni-jena.de/cat12/CAT12-Manual.pdf), the thickness data and folding data (gyrification and sulcal depth) were smoothed with 15 and 20 mm full-width at half-maximum Gaussian kernels, respectively.

### Statistical analysis

#### Voxel-based morphometry

##### Whole brain

Using the general linear model, voxel-by-voxel multiple regression analyses were performed across the whole brain to explore the association between psychopathic traits and GMV. The analyses tested for both positive and negative associations across total psychopathy, Factors 1 and 2, and each of the sub-facets scores (ASB, CA, EL, and IM) separately.

##### Region of interest

A priori regions of interest (ROI) were identified from previous literature regarding structures associated with psychopathic traits. Specifically, the ROIs were chosen based on brain regions implicated in the leading neurobiological models of psychopathy (Blair 2005; Kiehl 2006; Anderson and Kiehl 2012; Blair 2013) and the results of the De Brito et al. (2021b) meta-analysis. Bilateral anatomical masks were created using the Wake Forest University (WFU) Pick Atlas Toolbox (Maldjian et al. 2003) in SPM12 (ROIs: ACC, MCC, PCC, amygdala, caudate, Brodmann’s area 9/46, orbitofrontal cortex, putamen, insula). Furthermore, ROI analyses were also conducted using spheres with a radius of 10 mm centered at peak coordinates outlined in a recent meta-analysis of VBM studies investigating psychopathy and GMV (De Brito et al. 2021b) (ROIs: middle frontal gyrus, superior orbitofrontal, midcingulate gyrus, postcentral gyrus, caudate, inferior temporal gyrus, precentral gyrus. Also see supplementary materials Table S1 for exact coordinates and anatomical locations). The MarsBaR toolbox (Brett et al. 2002) was used within SPM12 to extract gray matter volume data and plot them to visualize any main or interaction effects. The MarsBaR toolbox works by calculating a single summary value (such as the mean (by default)) per participant across the significant voxels found within a specific ROI.

#### Surface-based morphometry

Similarly, general linear models were fitted in a vertex-wise whole brain analysis to test for associations between each of the surface-based metrics (thickness, sulcus depth, gyrification index) and total psychopathy, Factors 1 and 2, and each of the sub-component traits (ASB, CA, EL, and IM) separately.

For all main effect models, age and sex were included as covariates of no interest. A Factor 1 by Factor 2 interaction term was entered as a separate model. Furthermore, an interaction term between sex and psychopathy trait was included in the multiple regression analyses by setting up full factorial models. For interaction models, age was included as a covariate of no interest. Statistical maps were corrected for multiple comparisons at the voxel level using a familywise error rate (FWE) correction at *P* < 0.05. For completeness we report in the supplementary material (Fig. S2 and S3) the results of sub-facet (ASB, CA, EL, and IM) multiple regression analyses.

## Results

### Demographic, total intracranial volume and psychopathy scores

Participant demographic information and psychopathy scores are summarized in Table 1. The SRP-SF scores reported here are generally comparable with those presented in previous community samples (Seara-Cardoso et al. 2012), but unsurprisingly lower than scores from clinical and forensic samples (León-Mayer et al. 2015; Neumann et al. 2015; Debowska et al. 2018). There was a significant difference in TIV between males and females (*P* < 0.001, *d* = 1.76). The mean score for total psychopathy was 52 (SD = 13.7). There was no significant difference in total psychopathy, and subcomponent scores, between males and females (all *P*s > 0.05) except for erratic lifestyle where males scored higher than females (*P* = 0.01, *d* = 0.52).

**Table 1:** Sample characteristics: age, TIV and psychopathy scores.

	Total sample (*n* = 97)	Male (*n* = 45)	Female (*n* = 52)	
	M (± SD)	M (± SD)	M (± SD)	*P*-value
Age	27 (± 5.3)	27 (± 5.7)	27 (± 5.1)	0.93
TIV	1,497 (± 14.8)	1,601 (± 114.7)	1,408 (± 104.5)	<0.001
SRP-4				
Total psychopathy	52 (± 13.7)	54 (± 14.4)	50 (± 12.9)	0.17
Antisocial behavior	10 (± 2.8)	10 (± 3.4)	10 (± 2.2)	0.73
Callous affect	14 (± 4.2)	14 (± 4.4)	13 (± 4.1)	0.33
Erratic lifestyle	14 (± 4.1)	15 (± 4.3)	13 (± 3.8)	0.01
Interpersonal manipulation	14 (± 5.2)	15 (± 5)	14 (± 5.4)	0.52
Factor 1	28 (± 8.6)	29 (± 8.6)	27 (± 8.7)	0.39
Factor 2	24 (± 6)	25 (± 6.6)	23 (± 5.3)	0.06

*P*-values derived from *t*-tests.

### Neuroimaging results

#### Whole brain analysis

##### Voxel-based regressions

Across all participants (*n* = 97) there were no significant positive or negative correlations between psychopathy, or its subcomponent traits, and gray matter volume.

##### Surface-based regressions

**Fig. 1:**
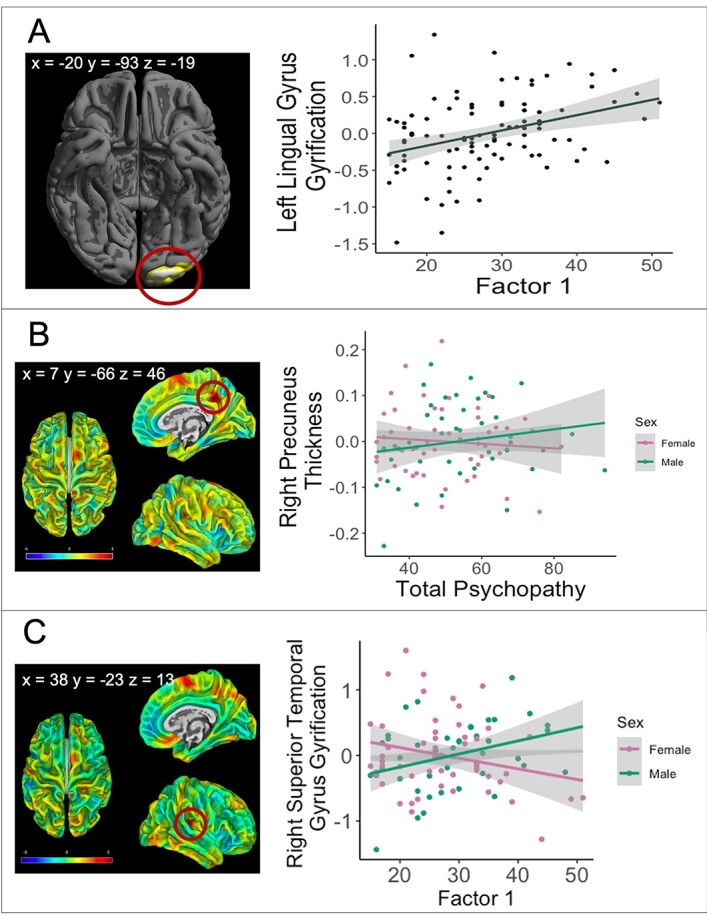
Cortical region and correlation depicting clusters in (a) the left lingual gyrus (*xyz* = −20 −93 −13) in which gyrification positively associated with Factor 1 scores (*P* = 0.03 FWE corrected, *k* = 66), after controlling for age, sex, and TIV and sex by psychopathy interaction effects in (b) the right precuneus (*xyz* = 7–66 46), in which overall psychopathy scores interacted with sex to predict cortical thickness (*P* = 0.036 FWE, *k* = 37) and (c) in the right superior temporal gyrus (*xyz* = 38, −23, 13) in which Factor 1 scores interacted with sex to predict gyrification (*P* = 0.008 FWE, *k* = 119) after controlling for age and TIV. Color bar represents *t*-values. Shaded ribbon represents 95% confidence interval. Results shown at *P* < 0.001, uncorrected for display purposes.

Higher Factor 1 scores were associated with increased gyrification in the left lingual gyrus within the occipital lobe (*x* = −20, *y* = −93, *z* = −13; *t* = 4.59; *k* = 66; *P* = 0.03 FWE corrected) (Fig. 1A).

Furthermore, total psychopathy scores interacted with sex to predict cortical thickness in the right precuneus (*x* = 7, *y* = −66, *z* = 46; *t* = 4.56; *k* = 37; *P* = 0.036 FWE) (Fig. 1B), such that psychopathy scores were positively correlated with cortical thickness in males, but negatively correlated with cortical thickness in females. Finally, Factor 1 scores interacted with sex to predict gyrification in the right superior temporal gyrus (*x* = 38, *y* = −23, *z* = 13; *t* = 4.99, *k* = 119; *P* = 0.008 FWE), such that Factor 1 scores were positively correlated with gyrification in males, but negatively correlated with gyrification in females (Fig. 1C).

#### ROI

All significant ROI results were derived from analyses using WFU Pickatlas anatomical masks. There were no significant associations between GMV and psychopathy traits when using the spheres generated using the meta-analysis peak co-ordinates.

##### Voxel-based regressions

**Fig. 2:**
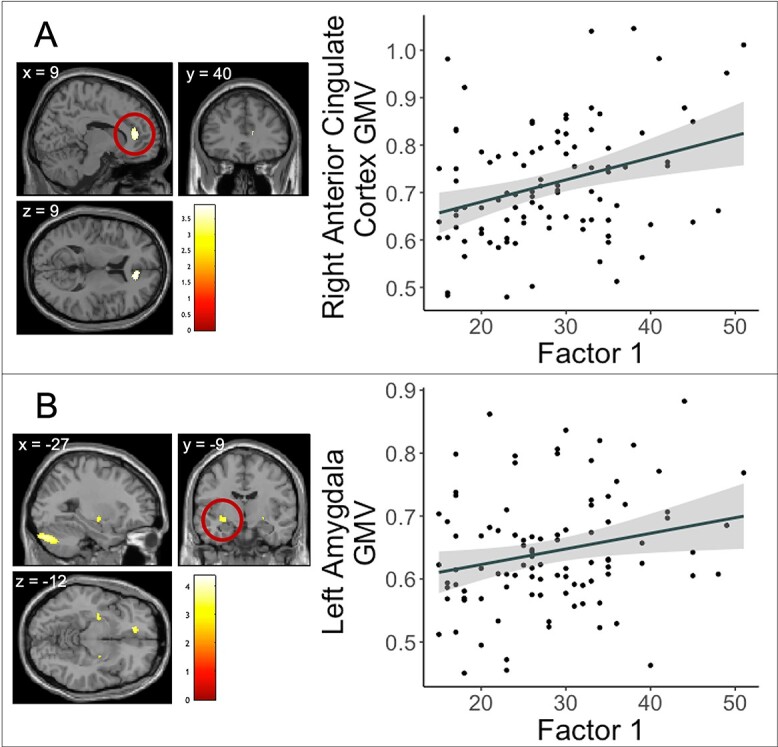
Brain region and correlation depicting clusters in the a) right anterior cingulate (*xyz* = 9 40 9) (Results shown at *P* < 0.001, uncorrected for display purposes) and b) the left amygdala/hippocampus (*xyz* = −27 −9 −12) (results shown are whole brain at *P* < 0.001, uncorrected for display purposes only) in which Factor 1 scores were positively associated with GMV (*P* = 0.014 FWE, cluster size = 306 and *P* = 0.034 FWE, cluster size = 1, respectively) after controlling for age, sex and TIV. Color bar represents *t*-values. Shaded ribbon represents 95% confidence interval.

Factor 1 scores were positively correlated with GMV in the right anterior cingulate (*x* = 9, *y* = 40, *z* = 9; *t* = 3.92, *k* = 306; *P* = 0.014 FWE) (Fig. 2A) and left amygdala (*x* = −27, *y* = −9, *z* = −12; *t* = 3.18; *k* = 1; *P* = 0.034 FWE) (Fig. 2B). Figure results are whole brain (not ROI) and shown at *P* < 0.001, uncorrected for display purposes only. The resulting cluster thus includes the amygdala, but also hippocampus, putamen, pallidum, and some white matter. However, the voxel that survived the FWE correction is located within the amygdala, on the border with hippocampus (please see supplementary material Fig. S1 for ROI mask and *P* < 0.05 FWE corrected cluster). As such, this finding will be referred to throughout the paper as amygdala/hippocampus

##### Surface-based regressions

**Fig. 3:**
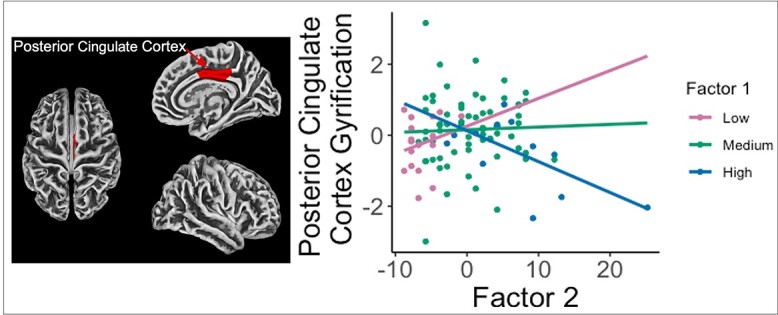
Cortical region and interaction effect depicting ROI, in which, Factor 2 scores were positively associated with gyrification in the right posterior cingulate cortex at low levels of Factor 1, but negatively associated with gyrification in the right posterior cingulate cortex at high levels of Factor 1.

A significant Factor 1 x Factor 2 interaction was observed for gyrification in the ROI analysis. Specifically, Factor 1 was moderating the association between Factor 2 and gyrification in the right posterior cingulate cortex (*P* = 0.045, FDR corrected; Fig. 3) such that, at high values of Factor 1 (> +1 SD), Factor 2 was negatively associated with gyrification in that region, while at low levels (< −1 SD) of Factor 1, the association between Factor 2 and gyrification was positive. One participant’s value was identified as an outlier. Removing the outlier did not alter the pattern of results (See Fig. S4 in supplementary materials) and the results remained significant.

## Discussion

Prior neuroimaging studies have provided evidence of structural abnormalities in mostly males with psychopathy from clinical and forensic settings. Yet, despite good evidence that psychopathy lies on a continuum and can be found to various degrees in the general population, it is unclear whether there is continuity of the psychopathic neural profile in the general population and, if so, whether sex or psychopathic factors moderate this neural profile. As such, this study sought to identify the neuroanatomical correlates of self-reported psychopathic traits using a dimensional approach in a mixed-sex community sample. The second aim was to determine if sex influenced the strength or direction of such associations. The final aim was to explore the interaction between psychopathy factors and brain structure to contextualize factor-related differences in brain morphometry.

The results of this study characterize the unique cortical morphology of psychopathy factors, by showing that Factor 1 scores, indexing the core affective and interpersonal features of psychopathy, positively correlated with gyrification in the lingual gyrus and volume of the anterior cingulate and amygdala/hippocampus. Furthermore, we show that sex moderates the association between psychopathy factor and two surface-based measures, gyrification and thickness. Finally, our findings suggest there may be distinct neurobiological characteristics of individuals that score high on both Factor 1 and Factor 2 dimensions in comparison to those who only score high on Factor 2.

The current study found a positive association between Factor 1 scores and gyrification within the occipital lobe, specifically within the lingual gyrus. This finding is in line with the only other study that has investigated gyrification in relation to psychopathy (Miskovich et al. 2018) where Factor 1 scores were positively correlated with gyrification bilaterally in the left and right occipital cortex. However, the findings from Miskovich et al. (2018) differ from our findings in that their result was bilateral, whereas, in the present study, the result was confined to the left hemisphere. Furthermore, the cluster observed by Miskovich et al. (2018) is larger and more dorsal than the one in the present study (in detail: Miskovich et al. (2018) left hemisphere MNI coordinates: *x* = −29, *y* = −90, *z* = 8, *k* = 1734, present study MNI coordinates: *x* = −20, *y* = −93, *z* = −13; *t* = 4.59; *k* = 66). This discrepancy could be due to differences in the nature of the samples. Miskovich et al. (2018) used male prison inmates, who may represent a more extreme version of psychopathy, whereas the current study used a community sample. Despite these differences, it is interesting and worth highlighting that there is some overlap between our cluster and the Miskovich et al. (2018) cluster given the differences in the study population.

Our finding is also in accord with previous VBM and SBM studies reporting structural abnormalities in the occipital cortex in individuals with psychopathy. For example, Bertsch et al. (2013) reported significantly reduced GMV in bilateral occipital cortex of antisocial offenders with high psychopathic traits. Ly et al. (2012) found that inmates with psychopathy had significantly thinner cortex in the left occipital cortex, in comparison with those without psychopathy. More recently, Miglin et al. (2021) found that Factor 1 scores were negatively associated with GMV in a cluster that included the left lingual gyrus. Furthermore, they observed a factor-level interaction effect in the left occipital lobe in a cluster that peaked in the lingual gyrus. This area has been implicated in the visual identification of neutral faces and facial expressions of emotions (Kesler et al. 2001; Kitada et al. 2010). The lingual gyrus forms part of the ventral visual pathway and houses the functional areas of the primary and extrastriate visual areas (V2 to V3) (Winawer and Witthoft 2017; Palejwala et al. 2021). To clarify the white matter connectivity and fiber bundle anatomy of the medial occipital lobe, Palejwala et al. (2021) performed diffusion spectrum imaging (DSI)-based tractography and gross anatomic dissection of the lingual gyrus (among other ROIs within the medial occipital lobe) and found that the medial occipital lobe is an extremely well-connected system with connections to several regions including the medial aspect of the fusiform gyrus, adjacent to the fusiform face area. Taken with findings from functional neuroimaging studies that have demonstrated that the lingual gyrus is involved in the early stages of facial processing (Luks and Simpson 2004; Fairhall and Ishai 2007), Palejwala et al. (2021) conclude that lingual gyrus may begin the task of processing facial information before the information is inputted to the fusiform face area in a bottom-up course of the ventral stream for more detailed processing to occur.

Additionally, support for distinct neural networks for facial (configural) recognition and emotional (feature) identification has been provided by functional MRI (Lobmaier et al. 2008) and PET (Rossion et al. 2000) studies. Notably, these authors showed facial featural processing selectively activated brain regions in the left hemisphere, including the left lingual gyrus (Lobmaier et al. 2008) suggesting a left hemisphere superiority for featural processing. Taken together the data suggest the brain regions involved in face perception are posterior to the fusiform face area and include the lingual gyrus. Therefore, although speculative, atypical lingual gyrus structural morphometry may be related to the impairments in recognizing emotional facial expressions that have been consistently linked to psychopathy (Wilson et al. 2011; Dawel et al. 2012). In sum, our study adds to an increasing body of SBM (Miskovich et al. 2018; Miglin et al. 2021) and VBM (De Brito et al. 2021b) data indicating that alterations within the occipital cortex might be important in understanding psychopathy; the fact that gyrification seems to be underlying the volumetric effect bolsters a neurodevelopmental conceptualization of psychopathy.

Region of interest analysis of gray matter volume and psychopathy factor scores demonstrated positive associations between GMV and Factor 1 scores in the right anterior cingulate cortex and left amygdala/hippocampal border. Previous studies have linked psychopathy to structural abnormalities in the anterior cingulate cortex (Johanson et al. 2020), which has been implicated in decision making and empathy related responses (Lavin et al. 2013). These authors mainly point to GMV reductions in these brain regions, however, Cope et al. (2012) also report a positive relationship between the ACC and psychopathy. The ACC has extensive functional and structural connections with regions known to be important for emotion related functions such as the amygdala (Etkin et al. 2006; Yang and Raine 2009; Stevens et al. 2011; Marsh et al. 2013). The amygdala is also a crucial element in neural systems underlying emotional processing and social behavior and is believed to be involved in stimulus-reinforcement learning (Murray 2007), the processing and response to emotional facial expressions (Adolphs et al. 1994; Deeley et al. 2006) and aversive conditioning (Selden et al. 1991; Büchel and Dolan 2000). Finally, the hippocampus is involved in episodic memory (Moscovitch et al. 2016) and lesions in this region have been linked to abnormalities in associative learning (Sziklas et al. 1998) and deficits in fear conditioning (Phillips and LeDoux 1992), both processes that have been shown to be impaired in psychopathy (De Brito et al. 2021a).

The locations where these positive associations occur, namely, the ACC and amygdala/hippocampus, are broadly consistent with brain regions noted in two leading theories of psychopathy (Blair 2005; Kiehl 2006; Anderson and Kiehl 2012; Blair 2013). Indeed, the central premise of Blair’s (2005) integrated emotion systems (IES) model is that amygdala dysfunction, or disruptions in its connected circuitry, gives rise to the core affective and interpersonal features of psychopathy. While the dysfunctional paralimbic system model (Kiehl 2006) posits that a core network of cortical and subcortical brain regions (including the ACC, PCC, OFC, amygdala, parahippocampal gyrus, insula, and superior temporal gyrus) are functionally and structurally altered in patients with psychopathy. Notably, Blair (2013) and Anderson and Kiehl (2012) hypothesize that people with psychopathy have reduced GMV in these regions, whereas the present study showed a positive association between psychopathy traits and GMV in the ACC and amygdala/hippocampus. Furthermore, although there is substantial functional MRI meta-analytical evidence that both regions are involved in psychopathy (Nickerson 2014; Poeppl et al. 2019; Deming and Koenigs 2020), the recent structural meta-analysis by De Brito et al. (2021b) did not find associations with psychopathy in either of these structures.

In relation to our second aim, a key finding was that sex acted as a moderator in the association between psychopathy factor and surface measures. Specifically, total psychopathy scores interacted with sex to predict cortical thickness in the right precuneus and Factor 1 scores interacted with sex to predict gyrification in the right superior temporal gyrus. In both regions, the SRP scores were positively correlated with surface measures in males, but negatively correlated in females. Previous authors have noted abnormal morphologies in the temporal cortex (De Brito et al. 2021b; Johanson et al. 2020; Kiehl 2006), in particular, the superior temporal gyrus region (Müller et al. 2008; Ly et al. 2012). The right superior temporal gyrus has been implicated in social cognition and the processing of social stimuli such as attention to emotion (Narumoto et al. 2001; Compton et al. 2003). As some aspects of these processes are known to be impaired in psychopathy (Drayton et al. 2018; De Brito et al. 2021a), our results could indicate that structural differences in the superior temporal gyrus may play a role in the pathogenesis of psychopathy. This finding is in line with Kiehl’s (2006) neurobiological model of psychopathy that identifies the superior temporal gyrus as part of a limbic/paralimbic network whose dysfunction is linked to psychopathy. The precuneus, serves as a core node of the default mode network and is engaged in numerous higher-order cognitive functions (Cavanna and Trimble 2006; Utevsky et al. 2014). Consistent with our findings, previous studies have reported structural alterations in the precuneus (mostly volumetric reductions) in individuals with psychopathy using male samples (Bertsch et al. 2013; Boccardi et al. 2011; Contreras-Rodríguez et al. 2015).

In contrast to earlier findings, however, sex differences emerged in the present study. This suggests there may be some shared, but some differential structural correlates of psychopathy in males and females, which may help us to understand the sexually dimorphic expressions of psychopathy outlined in literature (Tully et al. 2021). These results seem to be consistent with recent studies that have identified sex differences in the etiology of developmental trajectories of callous unemotional traits, which are considered a precursor of psychopathy and psychopathic personality in adults, from childhood to adulthood (Fontaine et al. 2010). Furthermore, these results from a community sample are broadly consistent with findings from more extreme samples (i.e. neurological patients) where there also seems to be evidence of sexual dimorphism in acquired sociopathy patients (de Oliveira-Souza et al. 2019). Given the novelty and the cross-sectional nature of our data a note of caution is due until they are replicated, but they provide additional support for sex-specific etiological pathways to psychopathy.

As for our final aim, we observed an interaction effect of Factor 1 and Factor 2 traits in gyrification in the right posterior cingulate cortex. Specifically, the association between Factor 2 and PCC gyrification depended on Factor 1 scores. Such that, as the Factor 1 score increased, the association between Factor 2 and PCC gyrification became more negative. The PCC is involved in self-referential processing and is a core node of the default mode network (Brewer et al. 2013). Neuroimaging investigations of psychopathy have repeatedly noted abnormal structural and functional connectivity in the PCC. Given its central role, much of this work focuses on the DMN and has linked psychopathy with attenuated functional connectivity in this network (Motzkin et al. 2011; Pujol et al. 2012; Juárez et al. 2013; Contreras-Rodríguez et al. 2015; Johanson et al. 2020). For example, Philippi et al. (2015) found distinct patterns of connectivity between the two psychopathy factors and reported positive associations between factor 2 scores and cortical connectivity between the medial prefrontal cortex and regions of the DMN including the PCC. While several VBM studies have found decreased GMV in the PCC in individuals with psychopathy (Johanson et al. 2020), much less is known about gyrification patterns.

Cortical folding patterns are believed to relate to underlying functional connectivity within neural networks so it is conceivable that abnormalities in gyrification patterns may reflect disrupted connectivity to extended cortical networks (Sasabayashi et al. 2021). Authors have speculated that increased local gyrification is an indicator of increased short-range connectivity and reduced long range connectivity (Dauvermann et al. 2012). The present findings provide some support for the premise that dysfunctional connectivity and the resulting abnormal cortical network dynamics may be related to the cognitive abnormalities in psychopathy. While speculative, atypical structures and dysfunction within cortical networks (i.e. diminished deactivation of the default mode network, hypersensitivity to task-based or environmental information or deficiencies switching between networks) could lead to competition for attentional resources (Freeman et al. 2015; Anderson et al. 2018; Deming and Koenigs 2020) and disrupt the normal-processing of socio-emotional information, which may, in turn, contribute to mood instability and behavioral characteristics (e.g. impulsivity, manipulation) commonly associated with psychopathy.

The interaction effect of Factor 1 and Factor 2 traits provides evidence that those who score high on both psychopathy factors may be neurobiologically distinct from those who score high on only one factor. This notion is in accord with a key finding from the meta-analysis by De Brito et al. (2021b) who also noted that structural differences (specifically, decreased GMV) in certain regions, including the PCC, were associated with increased scores on both factors whereas reductions in other regions were unique to only one factor. However, our finding is somewhat contrary to that of Miglin et al. (2021) who found that the association between Factor 1 traits and GMV in cortical and subcortical structures depended on Factor 2 scores. As such, further work is required to investigate the distinct and interactive factor-related differences in cortical structure.

It is worth noting that we generally found positive associations between psychopathy and volume- and surface-based metrics, especially when considering the male findings. This seems at odds with most of previous work, which points to volumetric reductions (Johanson et al. 2020; De Brito et al. 2021b) in males with psychopathy and negative associations between surface-based measures and psychopathic traits (Howner et al. 2012; Ly et al. 2012; Miskovich et al. 2018). A possible explanation for this notable difference lies in the fact that we used a sample of well-functioning adults from the community rather than a forensic or clinical population. Indeed, other studies also using community/non-incarcerated populations (Glenn et al. 2010; Cope et al. 2012; Vieira et al. 2015) have reported volumetric findings in the same direction as ours. Furthermore, in the present study, more than half of the participants were female, whereas, as previously outlined, most of the psychopathy literature contains male samples. Finally, this inconsistency could be attributed to the use of a non-Western sample. In future, a greater focus on discrete sample characteristics could produce interesting findings that account for the structural and functional differences that are present in the current psychopathy literature.

From a neurodevelopmental perspective it is worth highlighting that the majority of SBM findings related to gyrification patterns. Gyrification is a somewhat understudied surface-based metric, which describes the characteristic folding of the human cerebral cortex, which occurs as the disproportionately expanding brain is constrained by the cranial cavity (Hofman 1989). The process results in dense and efficient wiring that supports optimum neural activity (White et al. 2010). It has been proposed that investigating gyral patterns may be of interest when characterizing the specific morphology of neurological disorders for several reasons. Firstly, the formation of gross folding patterns occurs primarily during fetal development (Armstrong et al. 1995; White et al. 2010) and remains relatively stable with only some changes occurring during adolescence and into early adulthood (White et al. 2010; Hogstrom et al. 2013). As such, gyrification reflects fetal and early postnatal cortical development processes such as synaptic pruning and experience-related reshaping (Hogstrom et al. 2013; Zilles et al. 2013) and therefore may reveal the underlying biological processes of normal or abnormal cognitive functioning (Schaer et al. 2008). Furthermore, deviated cortical folding patterns have been demonstrated in several clinical disorders such as schizophrenia (Palaniyappan and Liddle 2012), autism spectrum disorder (Libero et al. 2019), bipolar disorder (Nenadic et al. 2015), major depressive disorder (Long et al. 2020) and Williams syndrome (Gaser et al. 2006). Lastly, while it is intrinsically related to surface area, gyrification provides an alternative biomarker that is distinct from other morphometric measures, such as cortical volume or thickness, that may provide a local measure of underlying pathology and represent a vulnerability for psychopathy.

While most neuroimaging studies in psychopathy have examined differences in GMV, cortical thickness or folding patterns in isolation, given that mechanisms that drive atypical development in one metric are likely to also interfere with the development of others, the combined assessment of brain morphology using VBM and SBM represents another strength of our study and contributes to a more fine-grained understanding of the structural brain correlates associated with psychopathic traits.

Limitations of our study include the use of a brief self-report measure of psychopathic traits. Such a measure could be susceptible to deception and likely does not approximate the in-depth and exhaustive PCL-R. A note of caution is therefore due when comparing self-report results with those obtained using PCL-R. Future research could look to replicate the current findings using other measures of psychopathic traits such as the Levenson Self-Report Psychopathy Scale (Levenson et al. 1995) or Psychopathic Personality Inventory–Revised (Lilienfeld et al. 2005), or if research constraints permit, the use of a semi-structured interview such as the Psychopathy Checklist: Screening Version (Hart et al. 1995). Additionally, there were no associations found when exploring Factor 2 scores. The reason for this is not clear but it is possible that our nil findings in relation to the impulsive-antisocial component may be attributed to using a community sample, which cannot cover a wide enough spread across these scores. Furthermore, no other socioeconomic status information was recorded, which makes it difficult to accurately assess the socio-occupational functioning of the participants. However, all participants self-reported no history of neurological or psychiatric diseases and the SRP-SF scores reported here are comparable with those presented in previous community samples. Finally, the current study design, which uses a non-Western community sample, makes it difficult to compare results from different samples (i.e. Western and forensic/clinical participants) and does not allow for our results to be easily generalized to other populations. Despite this, studying psychopathy in samples drawn from different populations fills an important gap in the literature and allows for a more comprehensive characterization of psychopathy.

## Conclusions

The results from this surface- and voxel-based morphometry study suggest that, in non-Western healthy adults, higher Factor 1 scores are associated with increased gyrification in the occipital lobe and increased GMV in the right anterior cingulate and left amygdala/hippocampus. Furthermore, to our knowledge, this is the first study to test for, and demonstrate, that males and females show differences in cortical structure, in opposite directions, relative to their sex, in the right superior temporal gyrus and precuneus. Finally, by exploring factor-level interactions, we present distinct cortical gyral patterns in individuals who score high on both factors compared to those that score high in only one factor.

## Supplementary Material

S_Chester_CC_SupMaterial_bhac397
