# Supplementary material for: Cortical and subcortical grey matter correlates of psychopathic traits in a Japanese community sample of young adults: sex and configurations of factors’ level matter!
Source: Cereb Cortex. 2022 Oct 27;33(9):5043–54. doi: 10.1093/cercor/bhac397 (PMC10151884; doi:10.1093/cercor/bhac397)
Supplement: S_Chester_CC_SupMaterial_bhac397 [file s_chester_cc_supmaterial_bhac397.docx]

**Supplementary Material**

**Cortical and subcortical grey matter correlates of psychopathic traits in a Japanese community sample of young adults: Sex and configurations of factors’ level matter!**

Sally C. Chester, Tatsuyoshi Ogawa, Maki Terao, Ryusuke Nakai, Nobuhito Abe, Stephane A. De Brito

**Methods and Materials**

**ROI Statistical analysis**

| **Table S1**. Anatomical locations and coordinates from De Brito et al (2021b) meta-analysis used to create ROI spheres | | |
| --- | --- | --- |
| Anatomical location (Brodmann area) | Hemisphere | MNI Coordinates (x, y, z) |
| Middle Frontal Gyrus (BA 10) | Left | -24, 54, 20 |
| Superior orbitofrontal (BA 11) | Left | -14, 36, -16 |
| Midcingulate gyrus (BA 23/24) | Left | 0, -10, 46 |
| Precentral Gyrus (BA 6) | Left | -38, -4, 58 |
| Caudate | Left | -10, 22, 0 |
| Inferior Temporal Gyrus (BA 20) | Right | 60, -24, -24 |
| Postcentral Gyrus (BA 4) | Left | -44, -16, 48 |
| Precentral Gyrus (BA 6) | Left | -50, -4, 26 |

**Results**

Factor 1 scores were positively correlated with GMV in the left amygdala (x = -27, y = -9, z = -12; *t* = 3.18; k = 1; *p* = .034 FWE). Figure S1 depicts the significant voxel at *p* < .05 FWE corrected and the ROI anatomic mask used to generate the significant cluster


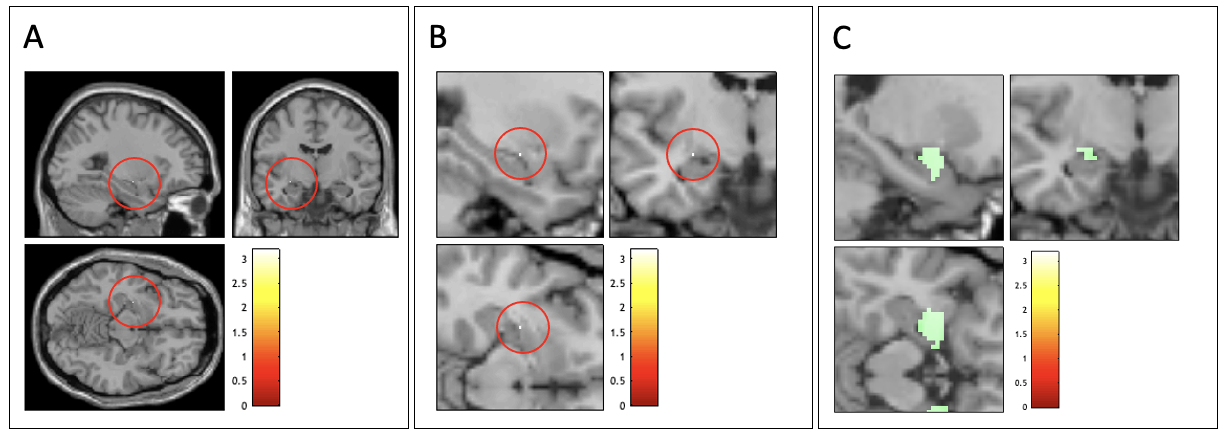


**Figure S1.** Brain region (a) (and zoomed in image (b)) depicting cluster in the left amygdala (xyz = -27 -9 -12) in which GMV positively associated with Factor 1 scores (*p* = .034 FWE corrected, k = 1) and c) the ROI mask used to generate this cluster.

*Surface-based regressions*

Higher interpersonal manipulation scores were associated with increased gyrification in in the left lingual gyrus (x = -20, y = -95, z= -18; *t* = 4.58; k = 39; *p* = .03 FWE corrected) and left calcarine sulcus (x = -11, y = -97, z = -15; *t* = 4.50; k = 19; *p* = .045 FWE corrected) (Figure S2).


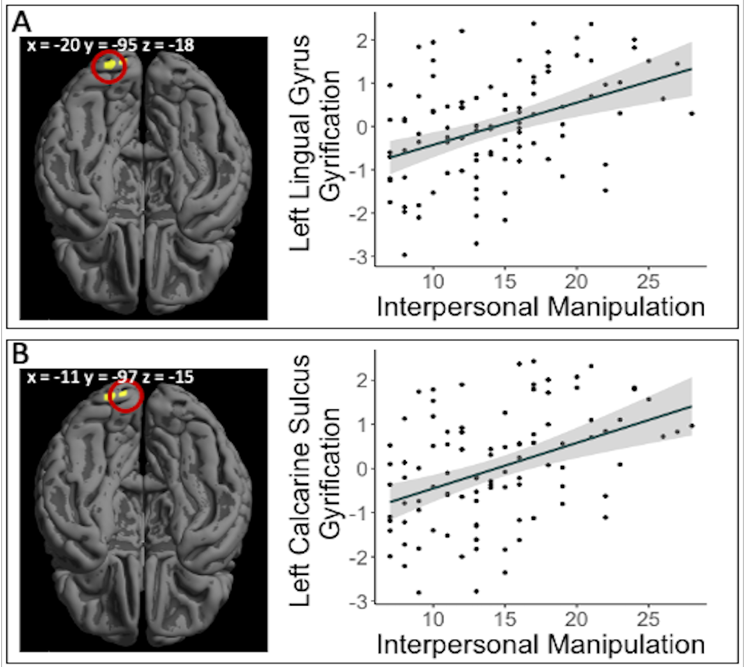


**Figure S2.** Cortical region and correlation depicting clusters in a) the left lingual gyrus (xyz = -20 -95 -18) in which gyrification positively associated with Interpersonal manipulation scores (*p* = .03 FWE corrected, k = 39) and b) the left calcarine sulcus (xyz = -11 -97 -15) in which gyrification positively associated with Interpersonal manipulation scores (*p* = .045 FWE corrected, k = 19) after controlling for age, sex and TIV. Shaded ribbon represents 95% confidence interval.

Interpersonal manipulation scores interacted with sex to predict gyrification in the right superior temporal gyrus (x = 36, y = -24, z = 13; *t* = 5.14, k = 147; *p* = .004 FWE), such that higher IM scores were positively correlated with gyrification in males, but negatively correlated with gyrification in females (Figure S3a).

Furthermore, interpersonal manipulation scores interacted with sex to predict cortical thickness in the right precuneus (x = 8, y = -44, z = 39; *t* = 4.53, k = 24; *p* = .039 FWE), such that females showed a positive, whereas males showed a negative association (Figure S3b).


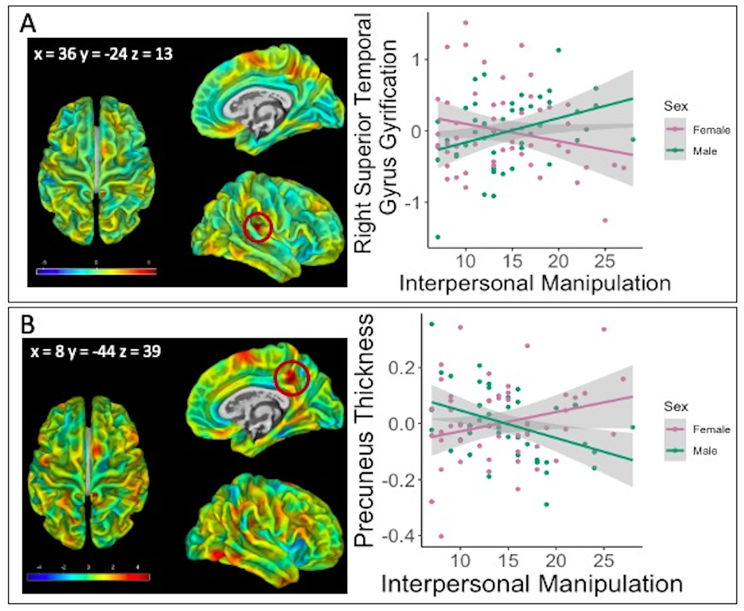


**Figure S3.** Cortical region and correlation depicting clusters in a) the right superior temporal gyrus (xyz = 36, -24, 13), in which interpersonal manipulation scores interacted with sex to predict gyrification (*p* = .004 FWE, k = 147) and b) the right precuneus (xyz = 8, -44, 39) in which interpersonal scores interacted with sex to predict cortical thickness (*p* = .039 FWE, k = 24) after controlling for age and TIV. Colour bar represents *t*-values. Shaded ribbon represents 95% confidence interval. Results shown at *p* < .001, uncorrected for display purposes.

A significant Factor 1 x Factor 2 interaction was observed for gyrification in the ROI analysis. Figure S4 depicts interaction effect with outlier included in analysis (Figure S4a) and outlier removed (Figure S4b).


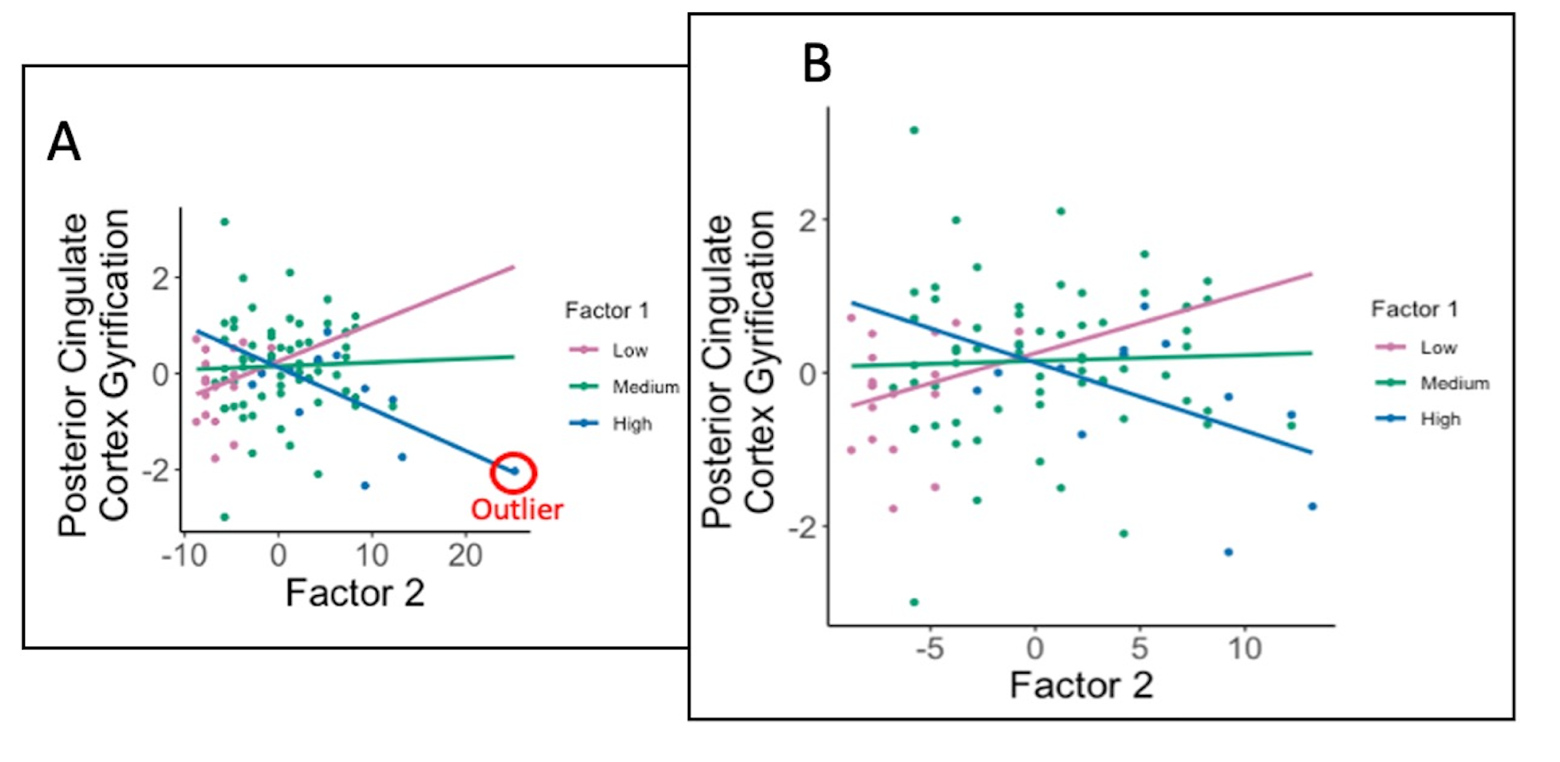


**Figure S4.** Cortical region and interaction effect depicting region of interest, in which, Factor 2 scores were positively associated with gyrification in the right posterior cingulate cortex at low levels of Factor 1, but negatively associated with gyrification in the right posterior cingulate cortex at high levels of Factor 1, with a) outlier included in analysis and b) outlier removed.
